# The predictive value of the nomogram model of clinical risk factors for ischemia–reperfusion injury after primary percutaneous coronary intervention

**DOI:** 10.1038/s41598-023-32222-2

**Published:** 2023-03-28

**Authors:** Zuoyan Wang, Jianjun Peng

**Affiliations:** Department of Cardiology, Beijing Shijitan Hospital, Capital Medical University, No. 10 Tieyi Road, Haidian District, Beijing, 100038 China

**Keywords:** Cardiology, Medical research, Risk factors

## Abstract

Ischemia–reperfusion injury is a risk factor for poor clinical prognosis in patients with ST-segment elevation myocardial infarction (STEMI). However, due to the inability to predict the risk of its occurrence early, the effect of intervention measures is still being determined. This study intends to construct a nomogram prediction model and evaluate its value in predicting the risk of ischemia–reperfusion injury (IRI) after primary percutaneous coronary intervention (PCI). The clinical admission data of 386 STEMI patients who underwent primary PCI were retrospectively analyzed. According to the degree of ST-segment resolution (STR), the patients were divided into the STR < 70% group (n = 197) and the STR > 70 group (n = 187). The least absolute shrinkage and selection operator (LASSO) regression method was used to screen out IRI's admission-related clinical risk factors. The R language software was used to construct and verify the IRI nomogram prediction model based on the above indicators. The peak troponin level and the incidence of in-hospital death in the STR < 70% group were significantly higher than those in the STR > 70% group (*p* < 0.01), and the left ventricular ejection fraction was significantly lower than that in the STR > 70% group (*p* < 0.01). Combined with the results of LASSO regression and receiver operating characteristic curve comparison analysis, we constructed a six-dimensional nomogram predictive model: hypertension, anterior myocardial infarction, culprit vessel, proximal occlusion, C-reactive protein (CRP) > 3.85 mg/L, white blood cell count, neutrophil cell count, and lymphocyte count. The area under the nomogram's receiver operating characteristic (ROC) curve was 0.779. The clinical decision curve found that the nomogram had good clinical applicability when the occurrence probability of IRI was between 0.23 and 0.95. The nomogram prediction model constructed based on six clinical factors at admission has good prediction efficiency and clinical applicability regarding the risk of IRI after primary PCI in patients with acute myocardial infarction.

## Introduction

Reperfusion therapy, including primary percutaneous coronary intervention and thrombolytic treatment, can restore the blood supply of ischemic myocardium in time and limit the infarct size^1^. Paradoxically, however, restoration of blood supply can cause additional heart damage and complications, known as ischemic-reperfusion injury (IRI)^2^. When coronary occlusion blocks the blood supply to cardiomyocytes, cardiomyocytes eventually become damaged or die. Still, restoring blood supply while the heart muscle is alive may also cause microvascular embolism^3^, increased calcium overload in cardiomyocytes^4^, excessive cardiomyocyte contracture ^5^, massive generation of oxygen free radicals^6^, leukocyte aggregation causing inflammatory damage^7^ and platelet activation^8^, and other harmful changes. The above-mentioned pathophysiological changes can cause abnormalities in the mechanical activity and electrophysiological function of cardiomyocytes, which clinically manifest as adverse clinical events such as arrhythmia^9^ and heart failure^10^.

Current clinical treatments for ischemia–reperfusion injury include ischemic preconditioning^11^, adenosine^12^, GPIIbIIIa receptor antagonists^13^, anti-oxidative stress^14^, and hypothermia therapy^15^. However, the validity of these methods and the normative approach to their application still need to be determined. Identifying high-risk patients with an ischemia–reperfusion injury early and applying the treatment mentioned above promptly and reasonably is an essential link to achieving effective clinical treatment of IRI.

Currently, the diagnostic methods for IRI include the level of electrocardiogram (ECG) ST-segment resolution^16^, myocardial blush grade^17^, the degree of increase and change trend of cardiac biomarkers, cardiac magnetic resonance imaging^18^, etc. However, the above indicators are obtained after IRI, and there is a lag in timeliness, so it is impossible to identify high-risk patients with IRI early enough to guide the timely adoption of cardioprotective strategies in clinical practice.

This study aims to investigate the early clinical risk factors of ischemia–reperfusion injury in patients with acute ST-segment elevation myocardial infarction (STEMI) after primary PCI and to construct a clinical prediction model to assist in the early risk assessment of IRI high-risk patients and improve clinical treatment strategy.

## Material and methods

### Study population

A total of 412 patients with STEMI who were admitted to the department of cardiovascular medicine at Beijing Shijitan Hospital and underwent primary PCI between January 2016 and October 2022 were retrospectively selected as the research subjects. Inclusion criteria: (1) Cardiac biomarkers exceeded 99% of the upper limit of reference values; (2) Sustained chest pain > 30 min or electrocardiogram showed ST-segment elevation of at least 0.1 mV in ≥ 2 adjacent leads; (3) Killip I–III grade on admission; (4) Onset < 12 h and successful primary PCI (5) Only revascularization was performed on the lesion vessel of the myocardial infarction during primary PCI. Exclusion criteria: (1) thrombolytic therapy before direct PCI; (2) cardiogenic shock on admission; (3) chronic total occlusion of non-infarction-related vessels; (4) previous myocardial infarction or coronary artery disease with a history of revascularization; (5) acute and chronic infectious diseases, hematopoietic abnormalities, autoimmune diseases, liver and kidney failure, and malignant tumor diseases. Finally, 386 patients were selected, including 314 males and 72 females, with a median age of 62.

### Collection of clinical data

Clinical data of patients were retrospectively collected through our hospital's clinical electronic medical record system and Hospital Information System (HIS), including: 1. demographic and disease-related data: gender, age, smoking history, and comorbidities; 2. admission echocardiogram results within 24 h; 3. Laboratory test results: including pre-intervention hematological parameters, such as white blood cells, lymphocytes, neutrophils, monocyte counts, hemoglobin, etc.; preoperative serum creatinine, preoperative C-reactive protein, peak troponin level (the troponin detection time is immediately after the operation, every 4 h after the procedure, and measured once every 48 h after reaching the peak, respectively); 4. Primary PCI intervention information: culprit vessel, ischemic infarction site, whether the occlusive lesion is located in the proximal end of the culprit’s vessel, postoperative TIMI blood flow classification, whether to use a suction device, use IABP or GPIIbIIIa receptor antagonists, whether to use stent therapy, stent type, and parameters, maximum balloon inflation pressure, etc.; 5. ST-segment resolution ratio (STR): Two cardiologists independently analyzed the ECG before and 60 min after the operation and selected the lead with the highest ST-segment elevation before the procedure to calculate the STR. The calculation formula is STR = (preoperative ST-segment elevation height—ST-segment elevation height 60 min after operation) ÷ ST-segment elevation height before operation. STR ≥ 70% means complete ST-segment regression, and STR < 70% means poor ST-segment regression.

### Statistical analysis

Statistical software SPSS 26.0 and R language (R 3.6.1) were used for data analysis, and measurement data were expressed as x ± s or M (P25, P75) according to whether they obeyed a normal distribution. The Mann–Whitney U test was used to compare the non-normally distributed data between the two groups. The independent sample t-test was used to compare the normally distributed data between the two groups. Categorical and graded data were expressed in case numbers, percentages, or rates. Categorical data were compared between the two groups based on the total sample size and the minimum theoretical frequency using the uncorrected Pearson chi-square test or Fisher's exact probability method. The least absolute shrinkage and selection operator (LASSO) regression method was used to screen the characteristic variables affecting STR. The "rms" program package in R language was used to construct the nomogram model and the calibration curve of the nomogram, and the "pROC" program package was used to draw the receiver operating characteristic (ROC) curve. The "caret" package was used to conduct Bootstrap self-sampling 1000 times to evaluate the prediction efficiency after internal validation, and the test level α = 0.05.

### Ethical statement

The Beijing Shijitan Hospital Ethics Committee, Capital Medical University approved the study protocol and written informed consent was obtained from all patients. All methods were performed in accordance with the Declaration of Helsinki.

## Results

### Characteristics of clinical data

**Figure 1:**
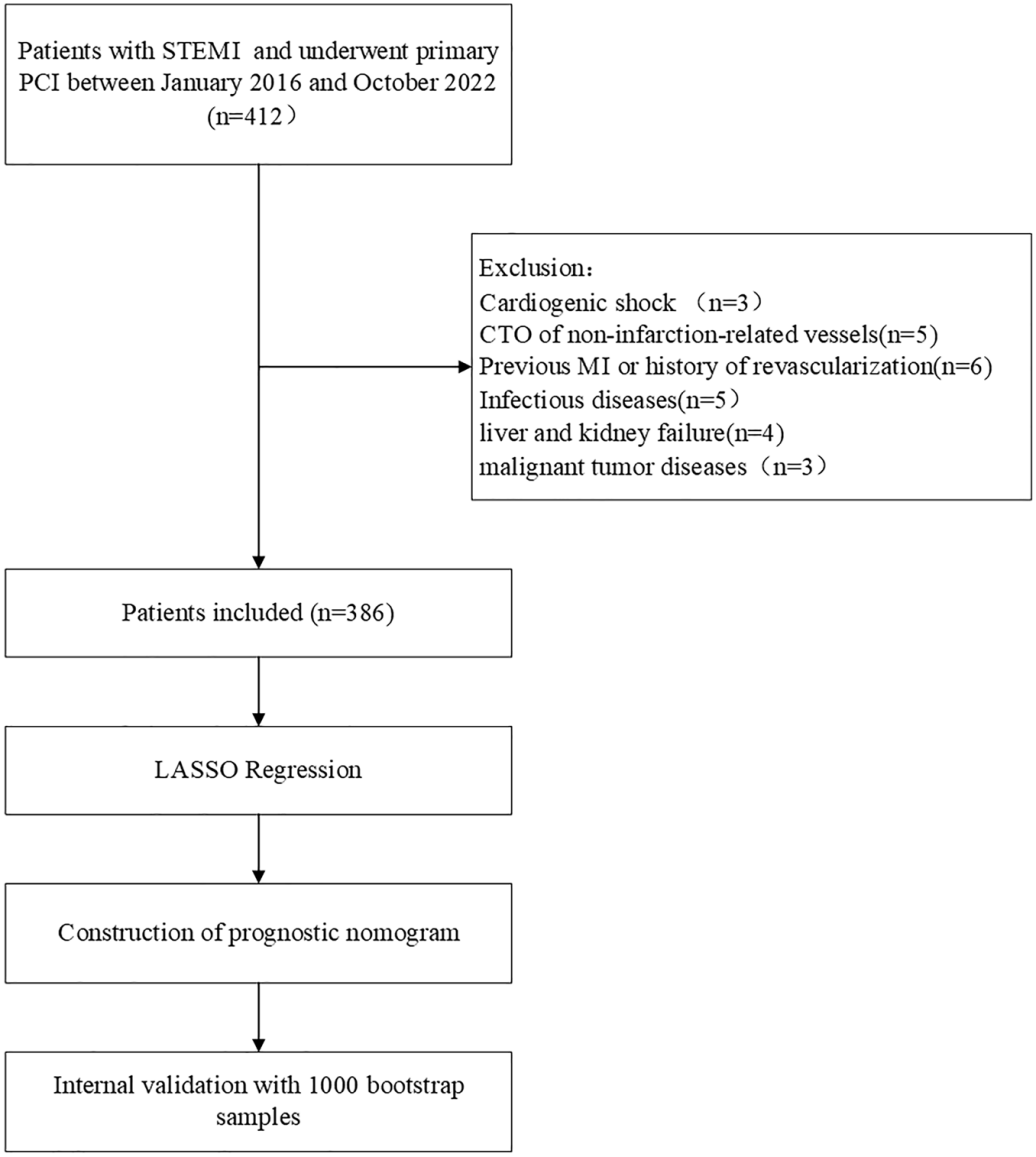
Study flowchart. STEMI, ST-segment elevation myocardial infarction; CTO, chronic total occlusion; MI, myocardial infarction; PCI, percutaneous coronary intervention; LASSO, least absolute shrinkage and selection operator.

Figure 1 illustrates the study flowchart. Among the 386 subjects, the incidence rate of poor ST-segment elevation recovery (STR < 70%) was 51.0% (197/386). Patients with STR < 70% had no significant difference in age, sex, diabetes mellitus, or smoking status compared with patients with STR ≥ 70%. Compared with patients with ≥ 70% STR, patients with poor STR had a higher proportion of hypertension. In the comparison of interventional treatment data, it was found that there was no significant difference between the two groups in terms of the time from symptom onset to balloon expansion, the application of a Glycoprotein IIb/IIIa antagonist, the application of a suction catheter, and the proportion of implanted stents. However, the proportions of anterior wall myocardial infarction, infarction-related vessel LAD, and proximal vascular occlusion in patients with poor STR were significantly higher than in patients with complete STR. The LVEF of patients with insufficient STR was significantly lower than that of patients with complete STR. Finally, patients with poor ST-segment resolution had significantly higher in-hospital mortality than patients with complete STR (5.1% vs. 0.5%, *p* = 0.007) (Table 1).

**Table 1:** Patient demographics and clinical characteristics.

Variables	Total (n = 386)	STR ≥ 70% (n = 189)	STR < 70% (n = 197)	*p*
Age,y ears	62.0 [53.0,73.0]	61.0 [52.0,70.0]	65.0 [53.0,75.0]	0.061
Male, n (%)	314 (81.3)	158 (83.6)	156 (79.2)	0.266
Diabetes mellitus ,n (%)	121 (31.3)	59 (31.2)	62 (31.5)	0.957
Hypertension, n (%)	227 (58.8)	100 (52.9)	127 (64.5)	0.021
Smoking, n (%)	166 (43.0)	80 (42.3)	86 (43.6)	0.792
Dyslipidemia, n (%)	224 (58.0)	113 (59.7)	111 (56.3)	0.493
Pain to balloon time, n (%)				0.991
< 3 h	334 (86.5)	164 (86.8)	170 (86.3)	
3–12 h	52 (13.5)	25 (13.2)	27 (13.7)	
Anterior Infarction, n (%)	191 (50.3)	70 (37.6)	121 (62.4)	< 0.001
IRA, n (%)				< 0.001
LAD	192 (49.7)	70 (37.0)	122 (61.9)	
LCX	38 (9.8)	25 (13.2)	13 (6.6)	
RCA	156 (40.5)	94 (49.8)	62 (31.5)	
Proximal Occlusion, n (%)	206 (55.1)	83 (45.9)	123 (63.7)	< 0.001
Glycoprotein IIb/IIIa antagonist, n (%)	229 (60.6)	108 (58.7)	121 (62.4)	0.465
Aspiration Device, n (%)	121 (32.0)	52 (28.4)	69 (35.4)	0.147
TIMI flow grade after PPCI, n (%)				0.005
≤ 2	82 (21.2)	29 (15.3)	53 (26.9)	
3	304 (78.8)	159 (84.7)	144 (73.1)	
Type of stent, n (%)				0.768
Bare mental stent	3 (0.8)	1 (0.5)	2 (1.0)	
Zotarolimus-eluting stent	112 (29.0)	60 (31.7)	52 (26.40)	
Sirolimus-eluting stent	190 (49.2)	89 (47.1)	101 (51.3)	
Everolimus-eluting stent	25 (6.5)	13 (6.9)	12 (6.1)	
LVEF, %, mean(± SD)	53.4 ± 8.3	55.6 ± 6.6	51.2 ± 9.1	< 0.001
In-hospital mortality, n (%)	11 (2.9)	1 (0.5)	10 (5.1)	0.007

*PPCI* primary percutaneous coronary intervention, *LVEF* ejection fraction, *TIMI* thrombolysis in myocardial infarction.

### Characteristics of laboratory examination

Compared with patients with > 70% STR, patients with poor STR had significantly higher peak troponin T, C-reactive protein level, total white blood cell, neutrophil, and monocyte counts. The level of lymphocytes in patients with poor STR was significantly lower than that in patients with > 70% STR. Patients with poor STR had higher D-dimer levels than those with > 70% STR, but the difference was insignificant. There were no significant differences in parameters such as hemoglobin level, hematocrit level, red blood cell count, platelet count, mean platelet volume, and platelet distribution width between the two groups. (Table 2).

**Table 2:** The admission laboratory results according to STR groups.

Variables	Total (n = 386)	STR ≥ 70% (n = 189)	STR < 70% (n = 197)	*p*
Peak troponin I, pg/ml	46,200.0 [13860.0,107,381.1]	25,780.0 [9750.0,79,120.0]	72,520.0 [22334.5,146,200.0]	< 0.001
C-reactive protein, mg/L	3.22 [1.49,7.97]	2.20 [1.28,4.60]	5.97 [2.16,13.40]	< 0.001
D-Dimer,ng/mL	118.0 [69.0,190.0]	98.0 [62.3,178.0]	132.0 [71.0,202.0]	0.068
White blood count, × 10^9^/L	9.51 [7.80,11.43]	9.27 [7.50,10.79]	9.90 [7.88,12.08]	0.023
Neutrophil count, × 10^9^/L	6.40 [4.80,8.40]	6.00 [4.65,7.70]	7.00 [5.10,9.40]	< 0.001
Lymphocyte count, × 10^9^/L	2.05 [1.37,2.78]	2.20 [1.49,3.10]	1.83 [1.20,2.50]	0.003
Monocyte count, × 10^9^/L	0.50 [0.39,0.69]	0.48 [0.39,0.64]	0.50 [0.39,0.70]	0.198
Neutrophil-to-lymphocyte ratio	3.29 [1.82,5.66]	2.66 [1.51,4.64]	3.94 [2.19,6.46]	< 0.001
Red blood cell	4.730 ± 0.531	4.733 ± 0.510	4.727 ± 0.551	0.928
Hemoglobin, g/dL	148.0 [135.0,156.0]	147.00 [135.00,157.00]	148.0 [135.0,156.0]	0.838
Hematocrit	0.43 [0.40,0.46]	0.43 [0.40,0.46]	0.43 [0.40,0.45]	0.780
Platelet count, × 10^9^/L	213.0 [180.0,253.0]	214.0 [181.0,256.0]	213.0 [179.0,253.0]	0.722
Mean platelet volume, fL	9.947 ± 1.070	9.896 ± 1.056	9.997 ± 1.080	0.367
Platelet distribution width	13.9 [11.7,16.2]	13.3 [11.7,16.1]	14.2 [11.6,16.2]	0.714
Red blood cell distribution width	12.8 [12.4,13.4]	12.8 [12.4,13.3]	12.8 [12.4,13.4]	0.872
Mean corpuscular volume, fL	90.6 [87.8,93.2]	90.8 [88.1,93.7]	90.0 [87.7,92.8]	0.251
Mean corpuscular hemoglobin, pg	31.0 [30.0,32.0]	31.0 [30.0,32.0]	31.0 [30.0,32.0]	0.914
Mean corpuscular hemoglobin concentration, g/dL	341.0 [334.0,351.0]	340.0 [333.0,349.0]	343.0 [335.0,353.0]	0.129

### Determination of the optimal cut-off value of CRP and the neutrophil/lymphocyte ratio for predicting STR < 70%

**Figure 2:**
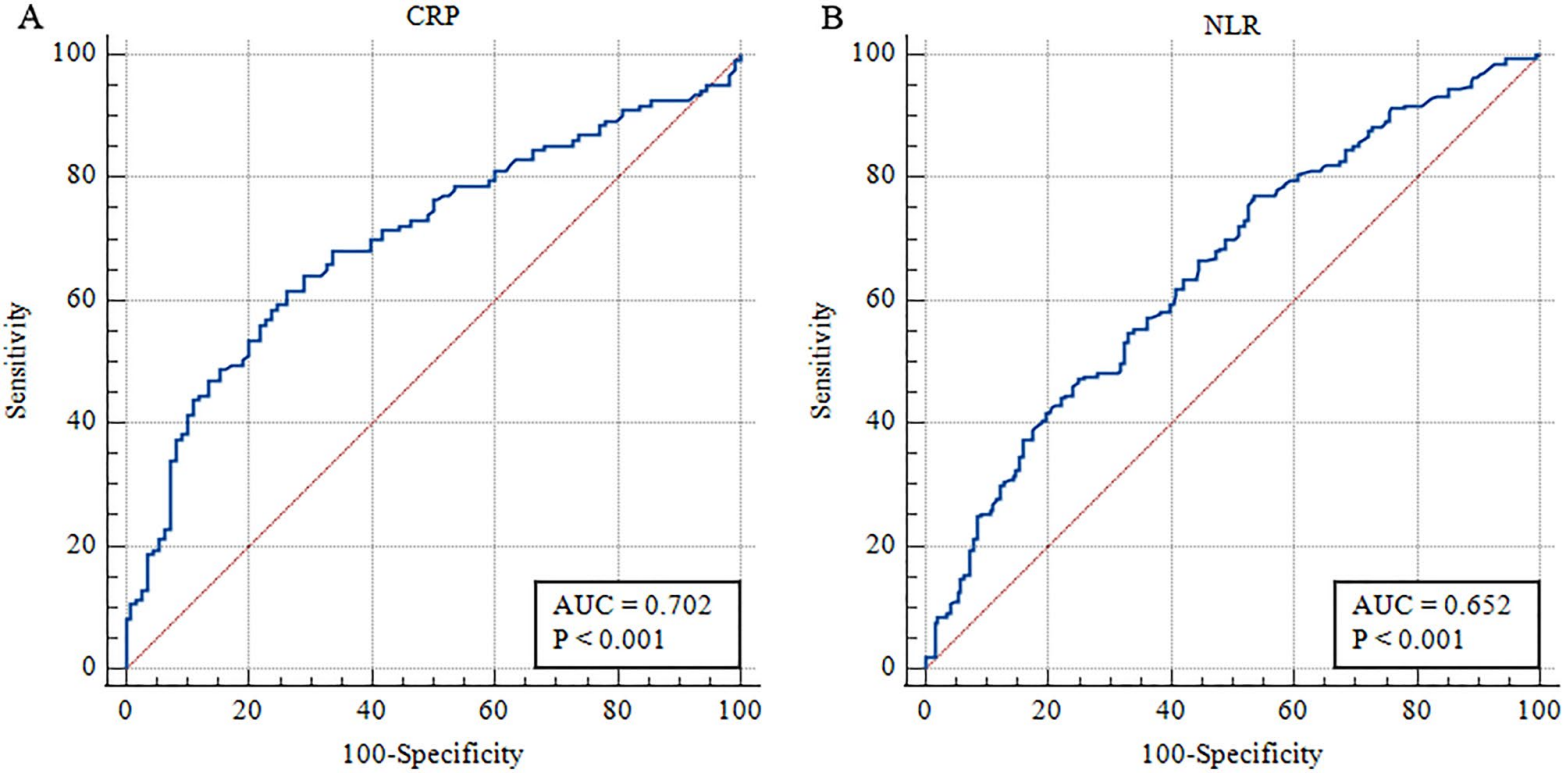
Optimal thresholds of C-reactive protein (CRP) and neutrophil to lymphocyte ratio (NLR) for predicting ST-segment resolution (STR) < 70%. (**A**) ROC curve of STR < 70% predicted by CRP; (**B**) ROC curve of STR < 70% predicted by NLR. AUC: area under the ROC curve.

We used receiver operating characteristic curves to explore the CRP and neutrophil/lymphocyte ratio values in predicting STR < 70%. The results showed that the area under the ROC curve (AUC) of CRP in predicting poor ST-segment regression was 0.702 with a 95% confidence interval (95% CI) of 0.638 to 0.760, and the optimal cut-off value was > 3.85 (Youden index 0.354, sensitivity 61.79%, specificity 73.64%). The AUC of NLR to predict poor ST-segment resolution was 0.652 (95% confidence interval 0.602 to 0.700), and the best cut-off value was > 2.17 (Youden index 0.2346, sensitivity 76.65%, specificity 46.81%) (Fig. 2).

### Screening of characteristic variables for the risk of adverse ST-segment regression after emergency PCI in STEMI patients

**Figure 3:**
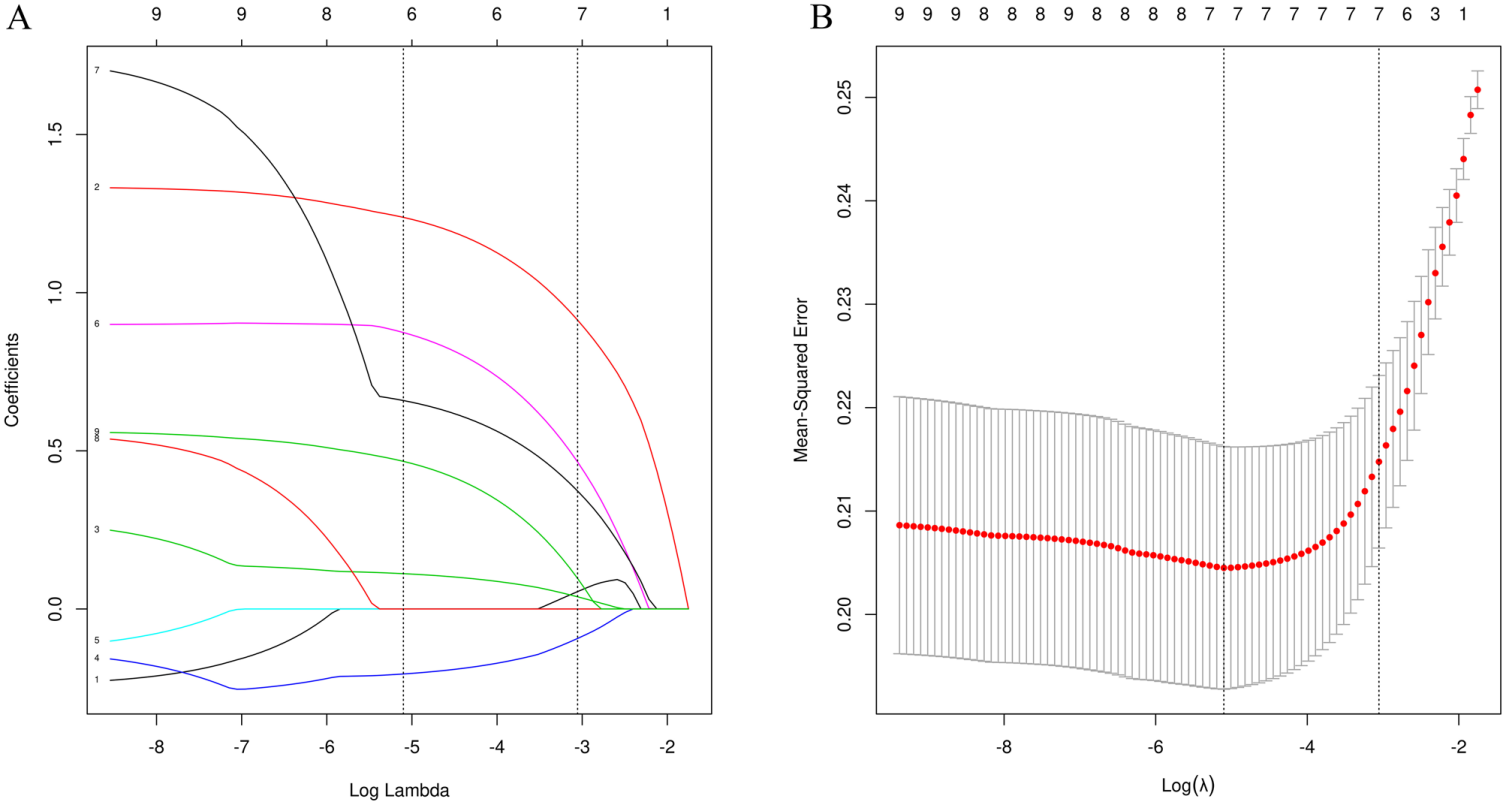
LASSO regression results for risk variables. (**A**) coefficient path diagram of risk variables; (**B**) cross-validation curve of LASSO regression.

Using LASSO regression, the nine admission and interventional variables with statistical significance (hypertension, anterior myocardial infarction, culprit vessel, proximal occlusion, CRP > 3.85 mg/L, white blood cell count, neutrophil cell count, lymphocyte count, neutrophil/lymphocyte ratio > 2.17, etc.) were subjected to dimensionality reduction processing to screen out further the characteristic variables of STR < 70% risk. Using tenfold cross-validation, the λ value when the cross-validation error value is the smallest is used as the optimal solution of the model. The variable names and variable numbers of the corresponding non-zero regression coefficients are counted at this time. LASSO regression results show that the λ value when the error is the smallest is 0.006, and there are seven variables corresponding to the non-zero regression coefficient, namely, hypertension, anterior wall myocardial infarction, proximal occlusive disease, CRP > 3.85 mg/L, neutrophil count, lymphocyte count, and NLR > 2.17 (Fig. 3). These seven variables are characteristic variables for predicting the occurrence of STR < 70%.

### Construction of a nomogram prediction model

According to the influencing factors found in Lasso regression, the "rms" package of R was used to construct the prototype of the nomogram for predicting the occurrence of ST-segment failure, and the scores of the seven indicators in the nomogram were added to obtain the total score. A vertical line is drawn along the corresponding score of the total score. The value on the horizontal axis of "STR < 70% risk" obtained is the risk value of poor ST-segment resolution after emergency PCI.

**Figure 4:**
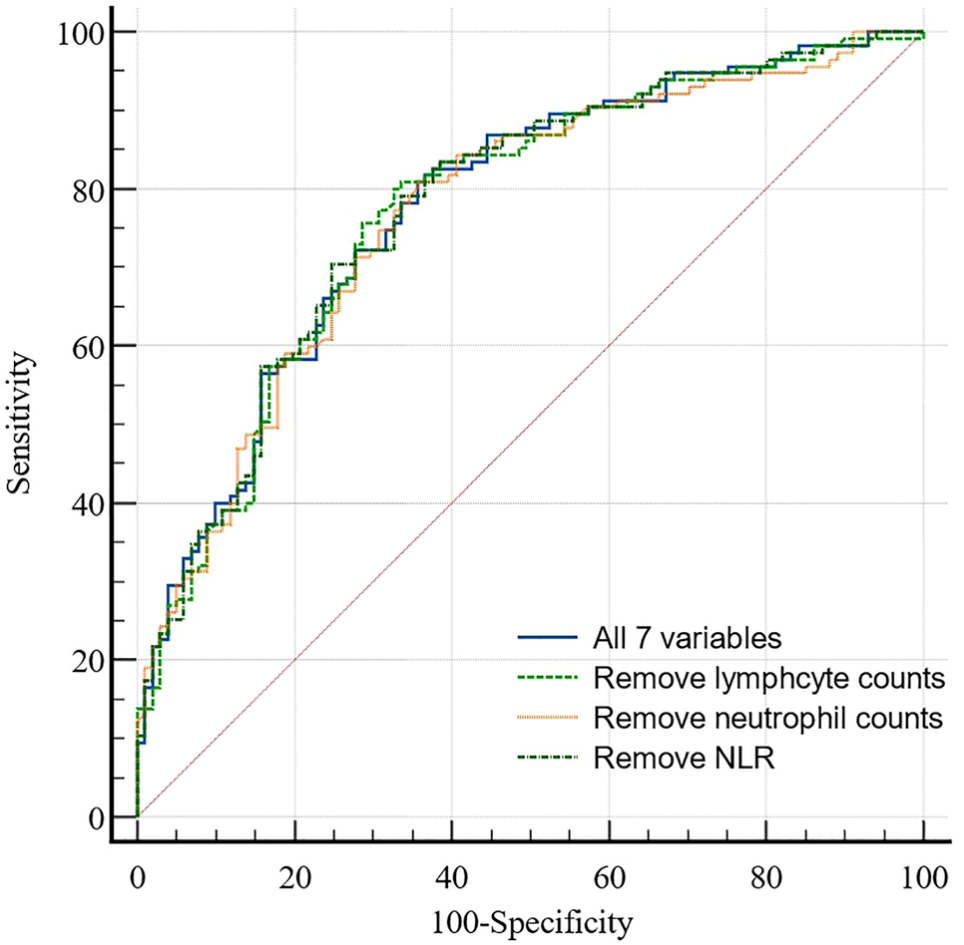
Comparison of ROC curves of nomogram models incorporating different variables to predict STR < 70%.

The AUC of the prototype nomogram model was 0.778 (95% CI 0.717–0.840). Considering that there is an interaction among neutrophil count, lymphocyte count, and NLR in the nomogram prediction model, and NLR ≥ 2.17 predicts that the AUC of STR < 70% is less than 0.70, we remove neutrophil count, lymphocyte count, and NLR, respectively, and form three improved models containing six variables. The results showed that the AUCs of the three improved nomogram prediction models were 0.775 (95% CI 0.714–0.829), 0.771 (95% CI 0.709–0.826), and 0.779 (95% CI 0.717–0.832), which were comparable to the 7-variable prototype. There was no significant difference in the AUC of the nomogram prediction model (Fig. 4).

**Figure 5:**
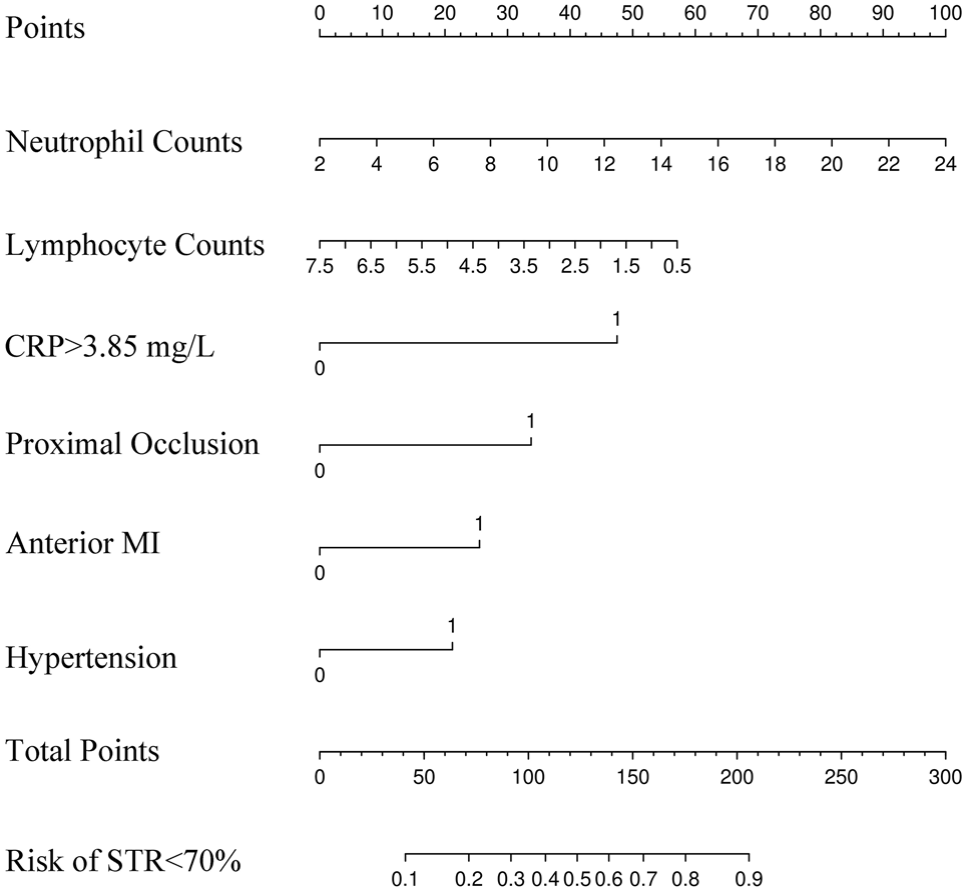
Nomogram model predicting STR < 70% after primary PCI.

**Figure 6:**
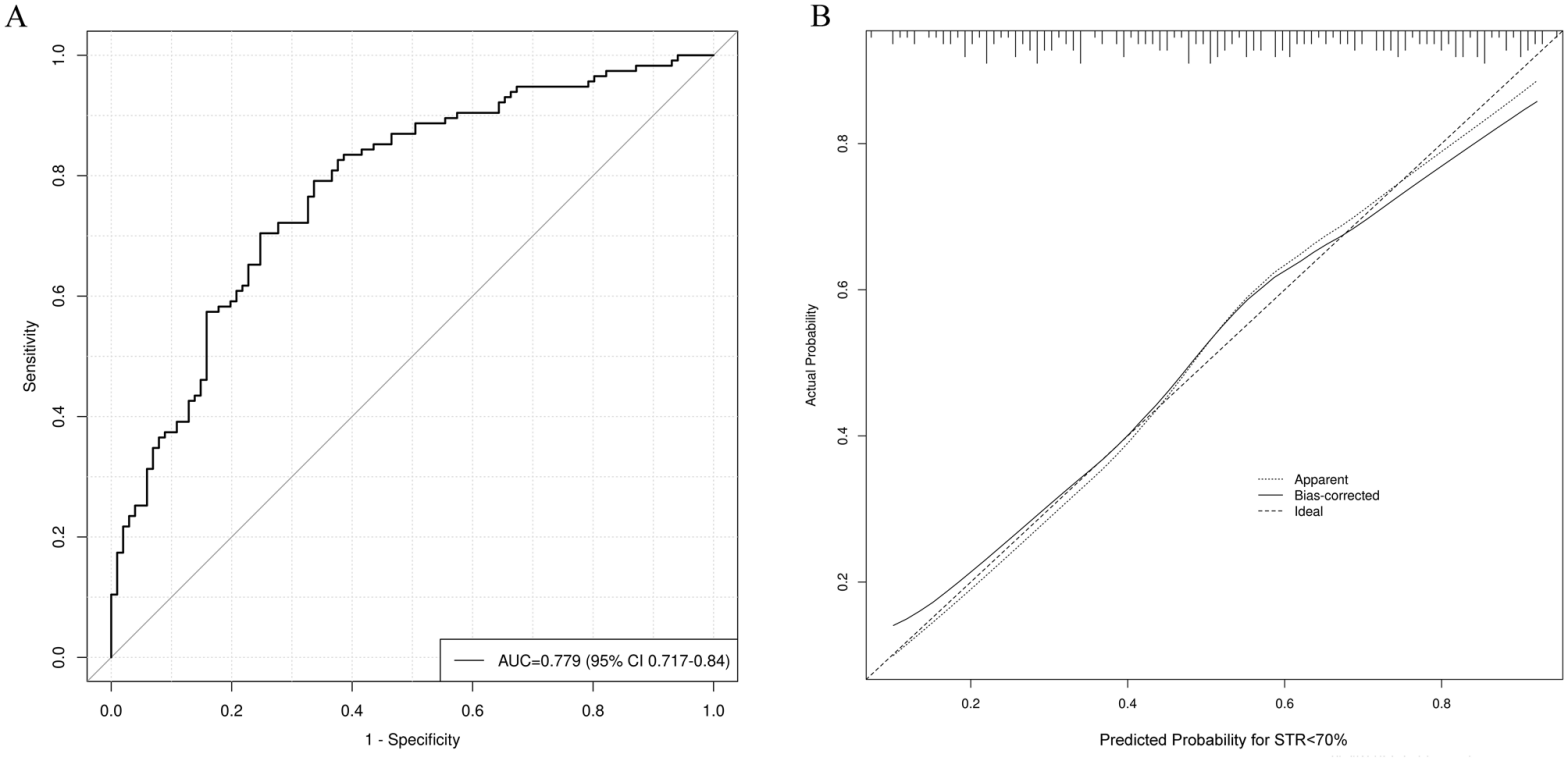
ROC curve (**A**) and calibration curve (**B**) of the nomogram model.

Therefore, we finally selected neutrophil count, lymphocyte count, hypertension, anterior wall myocardial infarction, proximal occlusive lesions, and CRP > 3.85 mg/L to construct a nomogram model (Fig. 5), and the AUC of the model was 0.779 (95% CI 0.717–0.832). The Hosmer–Lemeshow bias test suggested that the deviation between the risk prediction value of the nomogram and the actual value was not statistically significant (χ^2^ = 4.328, *p* = 0.826). Bootstrap was used to draw the internal calibration diagram, which showed that the risk-fitting curve of the nomogram was closer to the ideal curve, suggesting that the nomogram had a good calibration (Fig. 6).

### Analysis of the clinical applicability of the nomogram model

**Figure 7:**
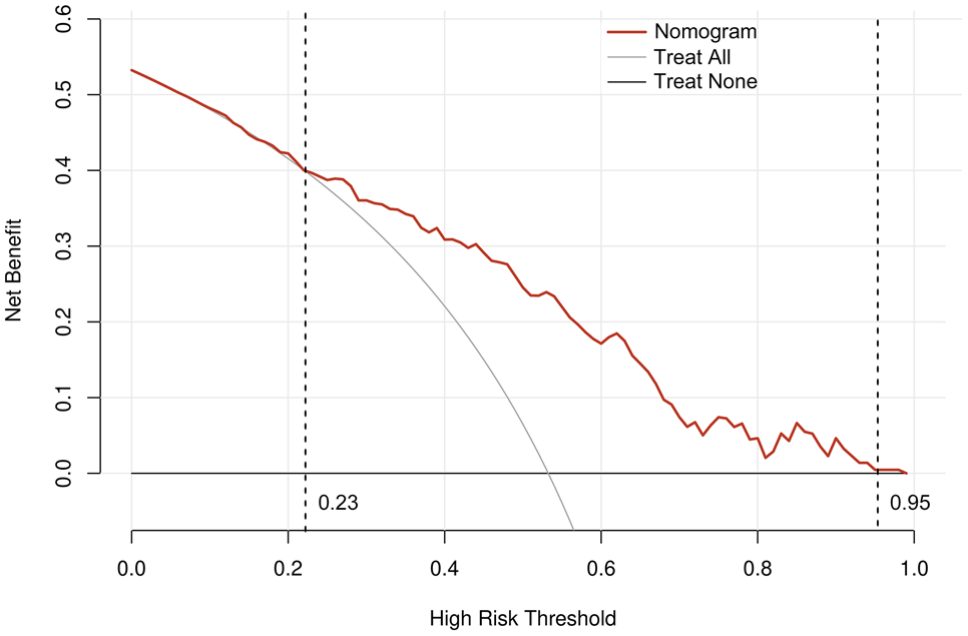
The decision-curve analysis (DCA) plot depicts the standardized net benefit of adopting the nomogram for predicting STR < 70%.

The predicted probability of the nomogram was used as the test variable, and the occurrence of STR < 70% in patients was used as the state variable to construct the clinical decision curve of the nomogram model. It can be seen from Fig. 7 that when the threshold probability of occurrence of STR < 70% in patients is between 0.23 and 0.95, the net benefit level of the application of the nomogram is higher than that of the "no intervention" and "all intervention" schemes. It is suggested that the nomogram has good clinical applicability.

## Discussion

Timely and active restoration of blood supply to the ischemic myocardium (reperfusion) and opening of infarction-related blood vessels are the most effective measures for treating myocardial infarction^19^. However, reperfusion itself is a double-edged knife. Restoring oxygen and nutrients to myocardial cells and saving the ischemic myocardium can also cause myocardial stun, no-reflow phenomenon, reperfusion arrhythmia, and even irreversible myocardial cell death, that is, "ischemia–reperfusion injury."^20^ From a pathological point of view, microvascular obstruction (MVO) and intramyocardial hemorrhage (IMH) are the main manifestations of reperfusion injury^21^. Evaluation methods for ischemia–reperfusion injury include biomarkers of myocardial injury, echocardiography, TIMI myocardial perfusion classification, positron emission tomography (PET/CT), quantitative single photon emission computed tomography (SPECT), cardiac magnetic resonance imaging (MRI), etc.

Studies have found that ST-segment resolution is significantly correlated with myocardial reperfusion at the microvascular level^22^, and can be used to evaluate reperfusion injury^16,23^. In this study, the proportion of patients with ST-segment resolution < 70% was 51.0%. These patients had a higher incidence of no-reflow, higher peak troponin levels, and in-hospital mortality compared with patients with ST-segment resolution > 70%. It is suggested that patients with ST-segment resolution < 70% have a clinically significant ischemia–reperfusion injury. This study used poor STR regression as an indirect criterion for judging ischemia–reperfusion injury. It was found that hypertension, anterior wall myocardial infarction, culprit vessel, proximal occlusion, CRP > 3.85 mg/L, white blood cell count, neutrophil count, lymphocyte count, neutrophil/lymphocyte ratio > 2.17, etc., were associated with ischemia–reperfusion injury.

Previous studies have also found that patients with hypertension and ventricular hypertrophy have increased susceptibility to ischemia–reperfusion injury. Hypertension and ventricular hypertrophy are more prone to myocardial cell apoptosis, fibrosis, arrhythmia, systolic dysfunction, and abnormal expression of gene level, thus aggravating the injury effect of ischemia–reperfusion^24^.

This study suggests that patients with anterior myocardial infarction and the culprit vessel LAD, especially proximal occlusion, are more susceptible to severe ischemia–reperfusion injury than patients with non-anterior myocardial infarction and the culprit vessel LCX or RCA (poor ST-segment regression). There are few studies on the correlation between different infarct locations and the culprit’s vessels and the severity of the ischemia–reperfusion injury. Some studies have found that the degree of ischemia–reperfusion injury in patients with an anterior wall infarction is more severe than that of an inferior wall infarction^25^. The response of patients with different infarct locations to anti-ischemia–reperfusion injury treatment is different, which is speculated to be related to the anatomical structure characteristics of different regions and the differences in collateral circulation status^26^. The no-reflow phenomenon is closely related to ischemia–reperfusion injury. This study found that the no-reflow incidence in patients with STR < 70% was significantly higher than in patients with STR ≥ 70% (26.9% vs. 15.3%, *p* = 0.005). This result is consistent with previous research^27–29^.

CRP level, white blood cell count, neutrophil count, lymphocyte count, neutrophil/lymphocyte ratio, etc., are important indicators to reflect the body’s inflammatory state, and the inflammatory response plays a vital role in ischemia–reperfusion injury^30,31^. CRP is an acute phase response protein. Animal studies have found that a large amount of CRP is deposited in the myocardial risk area of rat heart ischemia–reperfusion injury^32^. Excessive neutrophil infiltration can generate ROS and secrete chemokines while exerting phagocytosis on cell debris and matrix degradation^33^. Boag et al. found that decreased circulating T lymphocytes were associated with myocardial microvascular embolism and a poor prognosis in STEMI patients undergoing primary PCI^34^. Neutrophils and lymphocytes are markers of inflammatory persistence and regulatory pathways, respectively. As an indicator of systemic inflammatory status, the neutrophil-to-lymphocyte ratio (NLR) (calculated by dividing the neutrophil count by the lymphocyte count) is associated with adverse clinical outcomes in acute myocardial infarction^35^. Chen et al. found that NLR was positively correlated with myocardial injury in ACS patients undergoing emergency PCI, compared with patients with NLR < 2.76, patients with NLR > 2.76 had significantly increased myocardial injury markers and decreased systolic cardiac function^36^. Although understanding the pathophysiology of reperfusion injury can help identify potential therapeutic strategies, no treatment for reperfusion injury has been shown to improve clinical outcomes. The reasons for this include the inability to accurately predict the risk of ischemia–reperfusion injury and to give preventive or targeted treatment at the optimal time. Early identification of patients at the highest risk for reperfusion injury may be essential in bringing therapeutic approaches to clinical practice. In diagnosing and treating acute ST-segment elevation myocardial infarction, the average time from the first medical contact to the opening of epicardial culprit vessels to achieve reperfusion at the level of large vessels is 90 min^37^. During this period, the clinical data obtained by doctors is very limited, including the patient's demographic characteristics, electrocardiogram, blood routine, biochemistry, myocardial enzymes, etc., and coronary lesion characteristics obtained by coronary angiography. How to use these limited clinical data to establish a simple, fast, and accurate prediction model for the risk of ischemia–reperfusion injury to identify high-risk patients early is of great significance to improving the prognosis of STMEI patients.

A nomogram is a tool for transforming statistical models into visual graphics. It is simple, intuitive, and quantifiable. The nomogram has a good predictive effect and application value in predicting the risk of heart failure and death after primary PCI in patients with acute myocardial infarction (AMI). However, there is no nomogram model based on ischemia–reperfusion injury. This study found that indicators such as hypertension, anterior myocardial infarction, culprit vessels, proximal vascular occlusion, CRP > 3.85 mg/L, white blood cell count, neutrophil count, lymphocyte count, and neutrophil/lymphocyte ratio > 2.17 were associated with the risk of ischemia–reperfusion injury in STEMI patients. The clinical decision curve analysis showed that when the threshold probability of STR < 70% in patients was between 0.23 and 0.95, the net benefit of using the nomogram to guide treatment decisions was better than "intervention at all" or "no intervention at all.” The variables included in the prediction model are easy and fast to obtain. The time required for evaluation with model tools is short, which is conducive to clinical use and promotion.

The IRI prediction model constructed in this study has limitations, such as the lack of large samples and multi-center external verification. It still needs to be further verified and improved in future research. In this study, only STR was used as the criterion for judging IRI, which may have deviations from the actual changes of myocardial ischemia–reperfusion injury. Future analysis and exploration combined with myocardial imaging technology may further improve the accuracy of this nomogram in predicting the risk of IRI. Finally, since this study is retrospective, we did not routinely detect and collect indicators such as B-type natriuretic peptide (BNP)^38^, low-density lipoprotein cholesterol level^39^, and intravascular ultrasound (IVUS)-detected attenuated plaque^40^ that may be associated with ischemia–reperfusion injury in STEMI patients. In future studies, we will prospectively incorporate the above index parameters to explore the possibility of further improving the accuracy of the prediction model.

In conclusion, this study screened the clinical risk factors for admission of ischemia–reperfusion injury and constructed a nomogram prediction model. This model has specific predictive efficiency and clinical applicability and may help to identify STEMI patients with a high risk of ischemia–reperfusion injury early and provide corresponding prevention and treatment.

## Data Availability

The data supporting this study’s findings are available from the corresponding author upon reasonable request.
